# Vitamin B3 Provides Neuroprotection via Antioxidative Stress in a Rat Model of Anterior Ischemic Optic Neuropathy

**DOI:** 10.3390/antiox11122422

**Published:** 2022-12-08

**Authors:** Tu-Wen Chen, Po-Ying Wu, Yao-Tseng Wen, Tushar Dnyaneshwar Desai, Chin-Te Huang, Pei-Kang Liu, Rong-Kung Tsai

**Affiliations:** 1 Institute of Eye Research, Hualien Tzu Chi Hospital, Buddhist Tzu Chi Medical Foundation, Hualien 970, Taiwan; 2 Department of Ophthalmology, Kaohsiung Medical University Hospital, Kaohsiung Medical University, Kaohsiung 807, Taiwan; 3 Department of Ophthalmology, School of Medicine, Chung Shan Medical University Hospital, Chung Shan Medical University, Taichung 402, Taiwan; 4 Institute of Medical Sciences, Tzu Chi University, Hualien 970, Taiwan; 5 Doctoral Degree Program in Translational Medicine, Tzu Chi University and Academia Sinica, Hualien 970, Taiwan

**Keywords:** nonarteritic anterior ischemic optic neuropathy, vitamin B3, neuroprotection, oxidative stress, neuroinflammation, mitochondrial apoptosis

## Abstract

Supplementing with vitamin B3 has been reported to protect against retinal ganglion cell (RGC) damage events and exhibit multiple neuroprotective properties in a mouse model of optic nerve injury. In this study, a rat model of anterior ischemic optic neuropathy was used to assess the neuroprotective benefits of vitamin B3 (rAION). Vitamin B3 (500 mg/kg/day) or phosphate-buffered saline (PBS) was administered to the rAION-induced rats every day for 28 days. The vitamin B3-treated group had significantly higher first positive and second negative peak (P1-N2) amplitudes of flash visual-evoked potentials and RGC densities than the PBS-treated group (*p* < 0.05). A terminal deoxynucleotidyl transferase dUTP nick end labeling assay conducted on vitamin B3-treated rats revealed a significant reduction in apoptotic cells (*p* < 0.05). Superoxide dismutase and thiobarbituric acid reactive substance activity showed that vitamin B3 treatment decreased reactive oxygen species (*p* < 0.05). Therefore, vitamin B3 supplementation preserves vision in rAION-induced rats by reducing oxidative stress, neuroinflammation, and mitochondrial apoptosis.

## 1. Introduction

The most prevalent form of ischemic optic neuropathy is nonarteritic anterior ischemic optic neuropathy (NAION), which often manifests as acute unilateral vision loss without pain in people aged over 50 years [1,2]. The incidence of NAION has been reported to be approximately 23–103 per million people in the US and Taiwan [3,4].

A clear pathophysiology for NAION has yet to be identified, and no mechanism can be fully explained [5]. The optic nerve (ON) head’s infarction or inadequate blood flow is believed to be the cause of the disease, which is susceptible to small variations in blood supply, resulting in pathological changes [6,7,8]. Several factors contribute to NAION, including vasospasm, nocturnal hypotension, systemic vascular disease, and impaired microvascular autoregulation [3,9].

Until now, there has been no effective treatment for NAION. The ON and retina experience detrimental processes during ON ischemia, including the overinduction of oxidants and impairment of the regular antioxidative mechanism [8]. According to research by Birer et al., patients with NAION have more advanced oxidation protein products than healthy controls [10]. These investigations demonstrated a connection between acute ischemic optic neuropathy (AION) and high levels of oxidative stress. Although oxidative stress is a factor in the degeneration of retinal ganglion cells (RGCs) [11], its exact mechanism is yet unknown.

Niacin, often known as vitamin B3, is a precursor to the coenzymes nicotinamide adenine dinucleotide (NAD+) and nicotinamide adenine dinucleotide phosphate (NADP+), which are needed to enhance mitochondrial activities for the synthesis of adenosine triphosphate [12]. Therefore, the use of vitamin B3 to increase the NAD levels in various human tissues is becoming increasingly popular as a means of reducing age-related changes and neurodegenerative disorders. Recent studies showed that dietary supplements containing nicotinamide, an amide form of vitamin B3, have neuroprotective effects in glaucoma [13,14,15] and the retinas of diabetic rats [16]. In addition to RGCs, vitamin B3 can defend against age-related pathogenic processes in other cell types [17,18]. In glaucoma, a study reported that oral supplementation with vitamin B3 prevented RGC soma loss and RGC axonal loss that continued to support anterograde axonal in aged mice [19]. Based on these results, we suggest that the same neuroprotection of vitamin B3 can be seen on NAION by avoiding oxidative stress.

To the best of our knowledge, no prior research has looked into how vitamin B3 affects ON ischemia. Therefore, utilizing a rat model of AION, the purpose of this study was to investigate the neuroprotective benefits of vitamin B3 (rAION).

## 2. Materials and Methods

### 2.1. Study Design

The rats were randomly assigned to the sham group, the phosphate-buffered saline- (PBS-) treated group, and the vitamin B3-treated group (*n* = 30, each group). Laser treatment was administered to the rats in the sham group in the eyes that were not exposed to rose bengal (RB). The rats in the PBS-treated group were treated with an oral gavage of 0.1 mL PBS following the rAION induction, while the rats in the vitamin B3-treated group were administered an oral regime of 0.1 mL vitamin B3 (500 mg/kg/day) up to 28 days following the rAION induction. On day 2 after rAION induction, protein samples were collected for Western blotting analysis (*n* = 12, 24 eye globes). On day 7, the retina samples were analyzed for superoxide dismutase (SOD) activity and lipid peroxidation(*n* = 6, 12 eye globes). On day 28, the overall integrity of the visual circuit was determined using flash visual-evoked potential (fVEP) analysis (*n* = 6). On day 28, retrograde fluorogold labeling (*n* = 6) and in situ terminal deoxynucleotidyl transferase- (TdT-) dUTP nick end labeling (TUNEL) assays were used to detect RGC density and apoptosis. Sections of the ON were examined for the presence of macrophages infiltrating the ON 4 weeks after the ON infarction. The IHC techniques used samples sectioned from 12 eyeballs in each group.

### 2.2. Animals

90 outbred Wistar rats from a breeding colony weighing 150–180 g (7–8 weeks old) participated in the study (BioLASCO Co., Taipei, Taiwan). Each animal had a filter-top cage, and they were all given unlimited food. The temperature and humidity were both held at 23 °C and 55%, respectively, with a 12-h light-dark cycle. The Institutional Animal Care and Use Committee gave its approval to every animal experiment conducted at the Tzu Chi Medical Center. Animals were utilized in all experimental methods in compliance with the ARVO declaration on the use of animals in ophthalmic and vision research. Ketamine (100 mg/kg body weight) and xylazine (10 mg/kg body weight; Sigma, St. Louis, MO, USA) intramuscular injections were used to anesthetize all animals before the operations.

### 2.3. rAION Induction

Following sedation, the rats’ eyes were dilated using topical eye drops containing 0.5% tropicamide and 0.5% phenylephrine hydrochloride (Mydrin-P; Santen Pharmaceutical Co., Ishikawa, Japan), and local anesthesia was achieved using topical proparacaine hydrochloride ophthalmic solution (Alcaine solution 0.5%; Alcon, Puurs, Belgium). A 30-gage needle was used to administer 2.5 mM of RB in PBS intravenously (1 mL/kg of the animal’s body weight). Rats in the PBS- and vitamin B3-treated groups were next subjected to 12 pulses of a 532 nm argon green laser (MC-500 Multicolor Laser, NIDEK Co., Ltd., Gamagori, Japan), each lasting one second with a diameter of 500 μm and an output power of 80 mW, following the injection of RB. The rAION induction was considered successful when the ON head had a golden glow after laser exposure. Laser exposure without injection of RB was administered to rats in the sham group.

After the rats were kept on the heating pads at 37 °C to aid in their recovery, tobramycin 0.3% and dexamethasone 0.1% ophthalmic ointment (Tobradex ointment, Alcon, Puurs, Belgium) were applied to their eyes.

### 2.4. fVEPs

The fVEPs were measured using an E^3^ Module with a color dome system (Diagnosys LLC, Lowell, MA, USA). The skin covering the rats’ skulls was cut on day 28 following rAION induction to reveal the sagittal coordinates. Three electrodes were then implanted using a stereotactic device (Stoelting Co., Wood Dale, IL, USA) at the primary visual cortex and both hemispheres in the three coordinates anterior–posterior (AP), medial–lateral (ML), and dorsal–ventral (DV) (AP: −8.0 mm; ML: −3.0 mm). On the frontal cortex (AP: +3.0 mm), an electrode was anchored. The electrode in the frontal cortex served as a reference electrode, whereas the electrode in the primary visual cortex was active. Grounding of the system was achieved by connecting the corresponding electrode to the tail. A flash intensity of 30 cd/m^3^ and a flash rate of 1.02 Hz with no background illumination were used to measure fVEPs. An average of 64 sweeps was plotted on a graph. The amplitudes between the troughs of the first positive and the second negative waves (P1 and N2) were calculated.

### 2.5. Retrograde Labeling of Fluorogold

In our prior report, the retrograde labeling of the RGCs was covered [20]. The rats were put to sleep 21 days after receiving laser treatment, and the skin around their skulls was opened. The sagittal coordinates were marked (AP: −6.0 mm; ML: −1.5 mm; DV: +4.0 mm) using a stereotaxic apparatus, and perforated with a motorized dental drill. Using a Hamilton syringe, 2 μL of 5% fluorogold was injected into the superior colliculus (DV: +4.0 mm). The opposite hemisphere used the same process. Then, 7 days after administering fluorogold, the rats were sacrificed, and their eyeballs were removed and preserved in 10% formalin for 1 h. After fixing the retina on the slide, it was flat-mounted. In this study, fluorescent signals were captured from labeled RGCs using a 400 epifluorescence microscope (Axioskop; Carl Zeiss Meditec Inc., White Plains, NY, USA) with an excitation/barrier filter set (350–400 nm; barrier filter 515 nm) equipped with a digital camera (Axiocam MRm; Carl Zeiss Meditec Inc., NY, USA). The images were captured using AxioVision 4.0 software. The density of RGCs between 1 and 3 mm from the ON head was measured. Four randomly chosen locations (62,500 μm^2^) in the retina’s center were tallied, and the averages of those counts were used to determine the RGCs’ mean density. With the use of the Image Master 2D Platinum program, the RGC densities were measured (Version 7.0; GE Healthcare, Chicago, IL, USA).

### 2.6. Sample Preparation

Rats were sacrificed, and their eyes were harvested, on day 28 following rAION induction. After being fixed for 1 day in 4% paraformaldehyde, the samples were changed to 30% sucrose and kept at 4 °C until they dropped to the bottom as an indication of dehydration. The samples were embedded, then kept at −20 °C until sectioning. The retina and ON were cut into 30-μm-thick pieces in a cryostat chamber that was kept at −20 °C.

### 2.7. In Situ TUNEL Assay to Detect Apoptotic Cells

The ability of vitamin B3 to prevent RGC apoptosis was investigated using a TUNEL assay. Cutting the samples about 1–2 mm from the ON head allowed for the preparation of all frozen retinal slices. The retinal cross-section microscope slides were washed three times with 1× PBS for 5 min each. To limit the reagents required during the procedure, a liquid blocker was used after marking the samples. Retinal cross-sections were treated for 45 min with 100 mL of Proteinase K solution (20 g/mL), and then samples were incubated with 100 mL of equilibrium buffer for 10 min at room temperature. Using the Promega DeadEnd^TM^ Fluorometric TUNEL System Kit, a 50 μL reaction mix was created (DeadEnd™ Fluorometric TUNEL System, Promega Corporation, Madison, WI, USA). In a humid chamber, samples were incubated with this TdT reaction mix for 1 h at 37 °C. After three 5 min PBS-wash cycles, followed by mounting with Fluoroshield^TM^ with diamidino-2-phenylindole (DAPI) (1:100, Sigma, St. Louis, MO, USA), a confocal microscope was used to acquire images from the central retina to the midperipheral retina. By randomly selecting 10 high-power fields (HPF, ×200 magnification), we manually counted the RGC layer’s TUNEL-positive cells.

### 2.8. Immunohistochemistry

A marker for phagocytic macrophages and microglia is ectodysplasin 1 (ED1) [21]. Both intrinsic microglia and extrinsic macrophages are reacted by anti-ED1 antibodies [22]. The microslides were washed with PBS to remove the residual optimal cutting temperature compound. The boundaries were traced, and 3% bovine serum albumin (BSA) in PBST was used to block the tissues for 1 h at room temperature. The primary antibody ED1 (MCA341GA, Bio-Rad, Hercules, CA, USA) was prepared in a 1:100 concentration in 3% PBST. The tissue samples were then rinsed with 1× PBS three times for 5 min each after being incubated with the primary antibody for an overnight period. A 1:500 concentration of the secondary antibody was then prepared in 3% PBST. Tissue samples were rinsed with PBS for 5 min after being incubated with the secondary antibody for 1 h in a humid chamber. The tissue samples were mounted using Fluoroshield^TM^ with DAPI along with the DAPI counterstain. A fluorescence microscope was used to acquire images using the corresponding filters.

### 2.9. SOD Assay

Antioxidant enzyme activity was assessed using the SOD assay [23]. Retina samples were obtained, homogenized in radioimmunoprecipitation assay buffer at ice-cold temperatures, and centrifuged at 14,000× *g* for five minutes at 4 °C. Clean tubes were used for collecting the supernatant. The supernatant was then treated with a SOD activity test kit for 20 min at 37 °C (Colorimetric, ab65354, Abcam, Waltham, MA, USA). At 450 nm, an optical density measurement was carried out (*n* = 6).

### 2.10. Thiobarbituric Acid Reactive Substance (TBARS) Assay

The TBARS assay, which relies on the reaction of malondialdehyde, one of the byproducts of lipid peroxidation, with thiobarbituric acid, was used to measure the overall level of oxidized lipids [24]. In order to extract protein from the retinal tissue, RIPA buffer with protease and phosphatase inhibitors was utilized. The tissue was sonicated to facilitate protein extraction. After that, the samples were centrifuged at 1600× *g* for 10 min at 4 °C. The supernatant was gathered and stored in an ice bath. Then, protein concentrations were measured using the bicinchoninic acid (BCA) protein assay. All buffers and reagents were equilibrated at room temperature before use. After adding 100 μL of sample or standards to the corresponding tubes, 100 μL of sodium dodecyl sulfate solution was added and mixed thoroughly. Color reagent was applied to each vial at a volume of 4 μL. Then, the vials were submerged for 1 h in boiling water. After that, samples were incubated on ice for 10 min. The vials were then centrifuged at 1600× *g* at 4 °C for 10 min. After loading the contents of the vials into the ELISA plate reader (150 μL each well), the absorbance was measured at 530–540 nm in triplicate.

### 2.11. Western Blotting

The proteins were run in 1 NuPAGE MOPS running buffer on a gradient gel of 4–10% precast purchased from Invitrogen. The sample and 5 L of BLUelf prestained ladder were loaded in triplicate (GeneTex, Irvine, CA, USA). The proteins were transferred from the gel to a polyvinylidene difluoride (PVDF) membrane using Invitrogen’s iBlot2 dry transfer device and premade transfer apparatus. The membranes were then blocked in a solution of 1 TBST containing 5% nonfat milk. The primary antibodies were prepared using 5% BSA in 1 TBST. Using the iBright fl1000 imaging equipment, the membrane was first incubated in this ECL complex (Immobilon Western Chemiluminescent HRP substrate) for 3 min. The iBright Analysis software (Invitrogen, Carlsbad, CA, USA) was used to obtain and quantify the results.

### 2.12. Statistical Analysis

Data were expressed as mean ± standard deviation. Statistical analysis for data in all experiments was done using a non-parametric *t*-test (Mann–Whitney test). GraphPad Prism software was used for this purpose. A *p* value less than 0.05 was considered statistically significant.

## 3. Results

### 3.1. Vitamin B3 Preserves the Functional Visual Circuit

**Figure 1:**
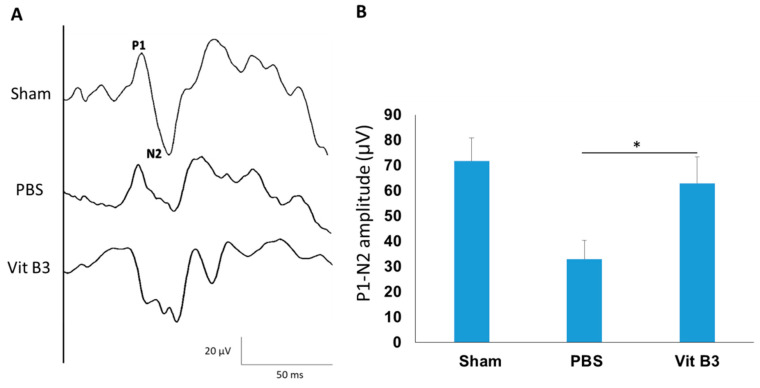
The fVEP assessments showed a significant improvement in the functional visual circuitry 28 days following vitamin B3 administration. (**A**) Each group included a representation of the fVEP wavelets. (**B**) The quantitative findings demonstrated that the P1-N2 wave amplitude was substantially greater in the vitamin B3-treated group than it was in the PBS-treated group (* *p* < 0.05; *n* = 6). Data were presented as mean ± standard deviation (SD). P1, the first positive peak; N2, the second negative peak.

Day 28 following the induction of rAION saw an analysis of the visual circuits (Figure 1A). The P1-N2 amplitudes in the PBS-treated group, the vitamin B3-treated group, and the sham group, respectively, were 71.69 ± 9.10 μV, 32.84 ± 7.62 μV, and 62.9 ± 10.37 μV (Figure 1B). In comparison to the control group, the PBS-treated group’s 2.18-fold significant decrease in the P1-N2 amplitude (*p* < 0.05) showed deterioration of the visual circuit after ON infarction, whereas the vitamin B3-treated group’s 1.9-fold significant increase in the P1-N2 amplitude (*p* < 0.05) showed preservation of the visual circuit after vitamin B3 treatment.

### 3.2. Vitamin B3 Treatment Promotes Survival of RGCs

**Figure 2:**
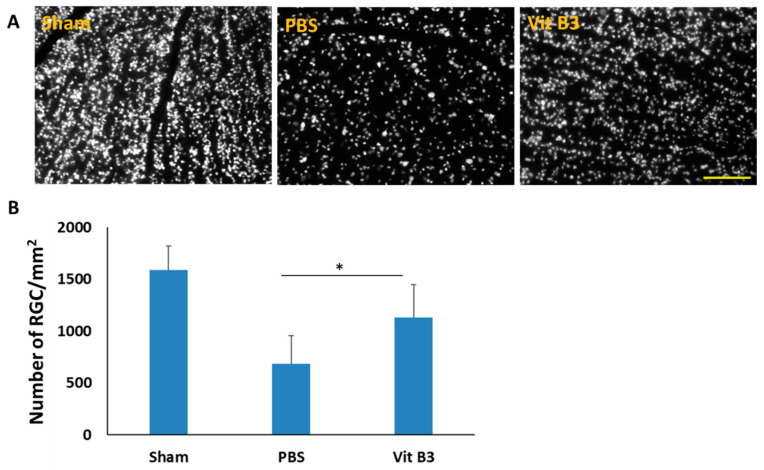
Vitamin B3’s impact on RGCs 28 days after an ON infarction. (**A**) Fluorogold-labeled RGC density that is typical for each group. (**B**) Following rAION induction, a bar chart revealed that the RGC density in the vitamin B3-treated group was considerably higher than that in the PBS-treated group (* *p* < 0.05; *n* = 6 per group; scale bar: 200 μm). Data were presented as mean ± SD.

A retrograde fluorogold labeling technique was used to detect RGC on day 28 after rAION induction (Figure 2A). RGC densities were 1547.6 ± 127.5/mm^2^, 647.2 ± 98.5/mm^2^, and 1086.7 ± 133.4/mm^2^ in the flat mounts of the retina for the sham group, PBS-treated group, and vitamin B3-treated group, respectively (Figure 2B). When compared to the sham group on day 28 following rAION induction, the RGC density in the PBS-treated group dramatically reduced 2.3-fold (*p* < 0.05), but it significantly rose 1.6-fold (*p* < 0.05) in the vitamin B3-treated group when compared to the PBS-treated group. A higher RGC density demonstrated the ability of vitamin B3 to rescue RGCs from ON ischemic injury.

### 3.3. Treatment with Vitamin B3 Inhibits RGC Apoptosis

**Figure 3:**
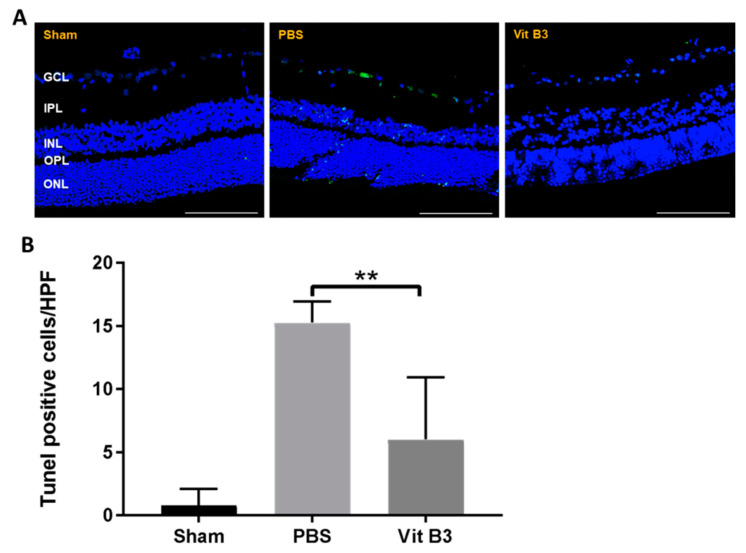
TUNEL assay analysis of RGC apoptosis in the RGC layer on day 28 following rAION induction. (**A**) Images of each group of retinal apoptotic cells after double staining. Blue staining was used to identify nuclei, whereas green staining was employed to identify apoptotic cells. (**B**) Calculation of the number of TUNEL-positive cells per HPF. The amount of TUNEL-positive cells was much less in the vitamin B3-treated group than it was in the PBS-treated group (** *p* < 0.01; *n* = 6 per group; scale bar: 100 μm). Data were presented as mean ± SD. GCL, ganglion cell layer; IPL, inner plexiform layer; INL, inner nuclear layer; OPL, outer plexiform layer; ONL, outer nuclear layer.

Apoptotic RGCs were detected using the TUNEL assay to further assess the RGC apoptosis in the retina (Figure 3A). In the sham group, the PBS-treated group, and the vitamin B3-treated group, respectively, there were 0.8 ± 1.3/HPF, 15.3 ± 1.7/HPF, and 6.0 ± 5.0/HPF of TUNEL-positive cells (Figure 3B). TUNEL-positive cells were 19.1-fold more prevalent in the PBS-treated group than in the sham group, whereas they were 2.5-fold less prevalent in the vitamin B3-treated group than in the PBS-treated group.

### 3.4. Vitamin B3 Prevents Macrophage Infiltration and Decreases Inflammation in the ON

**Figure 4:**
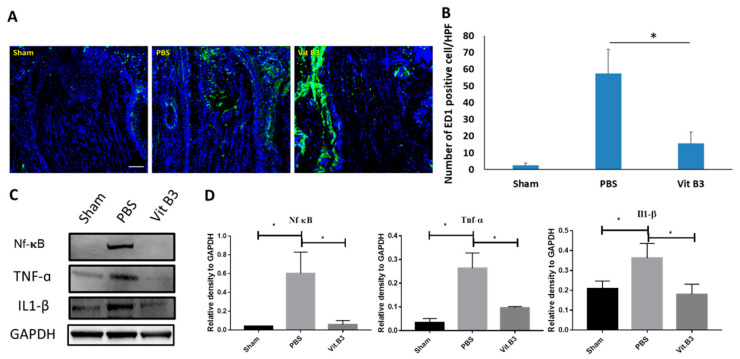
On macrophage infiltration in the ON and inflammation in the retina, vitamin B3 has an anti-inflammatory impact. (**A**) Pictures of the ONs in longitudinal sections showing ED1 staining. Green markers identified the ED1-positive cells. Blue labels identified the cell nuclei. (**B**) Counting the number of ED1-positive cells in each HPF. In comparison to the PBS-treated group, the vitamin B3-treated group had a considerably lower number of ED1-positive cells (* *p* < 0.05; *n* = 6 per group; scale bar: 100 μm). (**C**) Typical image of the day 2 following rAION induction Western blotting examination of the retinal material. (**D**) Measurement of the NF-B, IL-1, and TNF protein bands. Using the iBright imaging program, each value was calculated and normalized to glyceraldehyde-3-phosphate dehydrogenase (GAPDH). NF-B, IL-1, and TNF- expression were all significantly higher in the PBS-treated group than in the sham group, while they were all significantly lower in the vitamin B3-treated group than in the PBS group (* *p* < 0.05; *n* = 6 per group). Data were presented as mean ± SD.

The rAION induction caused severe inflammation of the ON. A large number of macrophages infiltrated the ON tissue as a result of the neuroinflammation [20]. ED1 antibody was used to stain these extrinsic macrophages, and DAPI was used to counterstain their nuclei (Figure 4A). The ED1-positive cell counts were 2.5 ± 0.8/HPF, 52.6 ± 14.2/HPF, and 15.7 ± 8.9/HPF in the control, PBS-treated, and vitamin B3-treated groups, respectively (Figure 4B). Vitamin B3 treatment reduced macrophage infiltration into the ON 3.4-fold (*p* < 0.05) after ON infarction.

In vivo, inhibiting astrocyte nuclear factor kappa B (NF-κB) can prevent ON damage and RGC loss [25]. Interleukin 1 beta (IL-1β) and tumor necrosis factor-alpha (TNF-α) are two cytokines that trigger inflammation and reactions that are regulated by NF-κB [26]. According to our immunoblotting results, vitamin B3 reduced the expression of NF-κB, IL-1β, and TNF-α, which may protect RGC from inflammatory damage (Figure 4C). In the PBS-treated group compared to the sham group, the levels of NF-κB, IL-1β, and TNF-α substantially rose 5.1-, 1.6-, and 2.5-fold, respectively. After ON infarction, vitamin B3 administration caused NF-κB, IL-1β, and TNF-α levels to dramatically decline 4.6-, 1.4-, and 2.1-fold, respectively, in comparison to the PBS-treated group (*p* < 0.05).

### 3.5. Vitamin B3 Treatment Increases Antioxidant Activity

**Figure 5:**
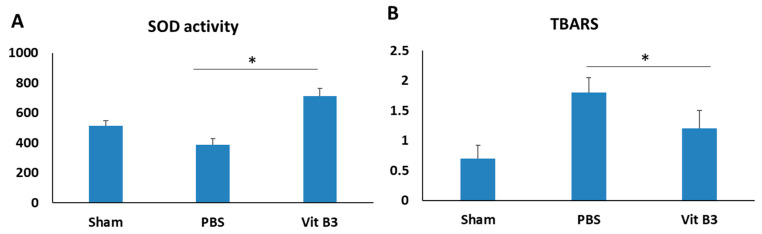
SOD activity and TBARS levels for oxidative stress were measured in the retina on day 7 following rAION induction. (**A**) In comparison to the PBS-treated group, the vitamin B3-treated group’s relative SOD activity was noticeably higher (* *p* < 0.05; *n* = 6 per group). (**B**) In comparison to the PBS-treated group, the relative TBARS in the vitamin B3-treated group was considerably lower (* *p* < 0.05; *n* = 6 per group). Data are expressed as the mean ± SD.

The ON infarction induced oxidative stress that degraded the antioxidant capacity of SOD [27]. Seven days after the infarction, the SOD activity in the PBS-treated group was 1.6-fold lower than that in the sham group (Figure 5A). On day 7 following rAION induction, the vitamin B3-treated group’s SOD activity was found to be 2.2 times higher than that of the PBS-treated group (*p* < 0.05) (Figure 5A).

A TBARS assay was used to measure lipid peroxidation, which represents oxidative damage in the retina after ON crush [24]. Seven days after rAION induction, the PBS-treated group’s level of lipid peroxidation increased 2.5-fold in comparison to the sham group, whereas vitamin B3 administration reduced the level 1.7-fold in comparison to the PBS group (*p* < 0.05) (Figure 5B).

### 3.6. Vitamin B3 Promotes Antioxidative Pathways in the Retina by Increasing Mitochondrial Metabolism

**Figure 6:**
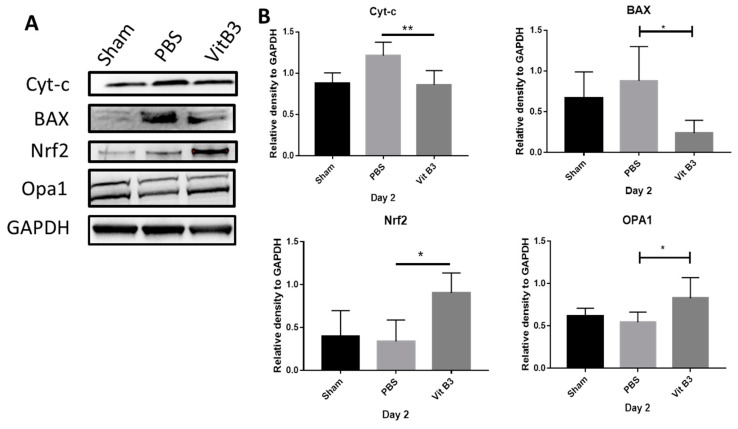
Analysis of the retinal expression of Nrf2, OPA1, cytochrome c, and BAX using immunoblotting. (**A**) Typical image of the day 2 following rAION induction Western blotting examination of the retinal samples. (**B**) Quantification of the Nrf2, OPA1, cytochrome c, and BAX protein bands. Each value was calculated with the help of the iBright imaging program and normalized to GAPDH. While the expression of Nrf2 and OPA1 was significantly higher in the vitamin B3-treated group than it was in the sham or PBS-treated group, the expression of cytochrome c and BAX was significantly lower in the vitamin B3-treated group than it was in the sham or PBS group (* *p* < 0.05; ** *p* < 0.01; *n* = 6 per group). Data were presented as mean ± SD.

In order to protect cells from oxidative damage, vitamin B3 increased the expression of nuclear factor erythroid-2-related factor 2 (Nrf2) [28]. Furthermore, vitamin B3 enhanced the expression of optic atrophy-1 (OPA1), cytochrome c, and Bcl-2-associated X (BAX), which were important in the modulation of mitochondrial function [29] and apoptosis [30], respectively. The mitochondria-mediated antioxidant potential was further assessed by immunoblot analysis on day 2. This indicated that vitamin B3 decreased cytochrome c and BAX levels by upregulating the antioxidant Nrf2 pathway. The upregulated Nrf-2 further regulated the mitochondrial fusion, which was evident from OPA-1 upregulation on day 2 (Figure 6A).

In the PBS-treated group compared to the control group, the levels of cytochrome c and BAX considerably rose 1.3- and 3.3-fold, respectively, while the levels of Nrf2 and OPA1 significantly dropped 0.74- and 0.8-fold, respectively (Figure 6B). The levels of cytochrome c and BAX significantly decreased after vitamin B3 therapy 0.86- and 0.51-fold, respectively, but the levels of Nrf2 and OPA1 dramatically rose 2.62- and 1.73-fold in comparison to the PBS-treated group (* *p* < 0.05; ** *p* < 0.01).

## 4. Discussion

During ischemic central nervous system (CNS) injury, inflammation, oxidative stress, and apoptosis frequently occur [31,32]. Ischemic CNS infarct at ON is followed by a significant amount of reactive oxygen species being produced, which result in severe neuroinflammation and progressive, prolonged loss of vision due to RGC death [33]. There is lack of knowledge with respect to the pathogenic mechanisms behind NAION, and no successful treatments are available for the condition, yet [5]. Therefore, preserving visual function and increasing RGC survival are important for developing new therapies for patients suffering from NAION.

In this study, vitamin B3 was shown to be neuroprotective in rAION. According to our data, vitamin B3 treatment after ON infarction reduced neuroinflammation and RGC death through the Nrf2-regulated antioxidative signaling pathway. Vitamin B3 significantly inhibited oxidative stress, inflammation, RGC apoptosis, and mitochondrial dysfunction after ON infarct.

Vitamin B3 treatment has been reported to prevent inflammation-related RGC degeneration in an ON injury mouse model [34,35]. Additionally, vitamin B3 treatment significantly protected against RGC damage events and exhibited multiple neuroprotective properties [36,37,38]. As Ischemic optic neuropathy has similar pathophysiology, we hoped vitamin B3 treatment regime would extend its neuroprotective benefits in this study. After ON infarction, vitamin B3 treatment significantly preserved the visual function (Figure 1A) and density of RGCs (Figure 2A). The TUNEL assay results revealed that vitamin B3 treatment inhibited RGC apoptosis in rAION (Figure 3A). Our findings suggest that vitamin B3 administration after ON infarction provides an antiapoptotic effect on RGCs.

Retinal ischemia is considered one of the most common features of ischemic retinopathy [39]. The vulnerable retina becomes more susceptible due to ischemia-induced oxidative stress [39]. Nrf2 plays an important role in protecting cells from oxidative damage in neurodegenerative diseases [28]. Studies have demonstrated that significantly increasing Nrf2 expression binds to antioxidant response elements (AREs) and increases NAD(P)H quinone dehydrogenase-1 (NQO1) and heme oxygenase-1 (HO-1) expression in response to oxidative insults [40]. The antioxidant (Nrf2/HO-1) pathway is stimulated by vitamins with antioxidant capacity, according to studies, indicating a crucial function in oxidative stress [41,42].

Our hypothesis is that vitamin B3 protects the rAION by activating antioxidant signaling through Nrf2. A number of antioxidant enzymes’ activities are increased by vitamin B3 through increasing the expression of Nrf2 and inducing nuclear translocation [43]. The movement of Nrf2 from the cytoplasm to the nucleus is aided by oxidative stress. The Nrf2 protein triggers the expression of a series of antioxidant proteins in the nucleus [44]. Additionally, the demonstrated increase in the enzymatic activity of SOD and decrease in lipid peroxidation of TBARS in the retina following rAION provide evidence for an enhanced antioxidant response by vitamin B3 supplementation (Figure 5). According to these results, vitamin B3 induces the Nrf2 protein level in the rAION-induced model, thus suppressing oxidative stress.

Our prior study suggested that therapeutic approaches in NAION must concentrate on the management of neuroinflammation, including prevention of macrophage infiltration, control of macrophage/microglia polarization, and decrease of pro-inflammatory cytokine production [21,45]. According to previous studies, ED-1-positive cells have been found in the ON during ischemic injury [46]. These cells enhance the transfer of microglia/macrophages to an activated form, contribute to the secretion of inflammatory cytokines, and activate inflammation [47]. The IF images indicate that reducing macrophage infiltration is localized to the ON by confocal microscopy with antibodies specific for ED1 (Figure 4A). According to Brambilla et al., in experimental optic neuritis, transgenic suppression of astroglial NF-κB reduces ON damage and RGC loss [25]. This shows that treating ocular neuritis with long-term NF-κB suppression yields favorable results [25]. Numerous studies have demonstrated that the administration of vitamins or their derivatives can prevent the NF-κB signaling pathway from being activated, and the impact this has on the linked downstream inflammatory cytokines, such as IL-1β and TNF-α, as well as their production [26]. The results of the immunoblot test show that the expression of NF-κB, IL-1β, and TNF-α seems to decrease after vitamin B3 administration. Vitamin B3 dramatically reduced NF-κB activation caused by rAION, which in turn decreased the generation of pro-inflammatory cytokines like IL-1β and TNF-α in vivo (Figure 4C). According to recent research, vitamin B3 regulates the advanced glycation end products (RAGE)/c-Jun N-terminal kinases (JNK)/NF-κB signaling pathway after mouse brain damage to reduce neuroinflammation (IL-1β and TNF-α) and neuronal death, restore memory impairment [48,49], and attenuate inflammation, and has been shown to promote RGC survival [50]. In this study, we also confirmed the anti-inflammatory effect of vitamin B3 treatment on the rAION-induced neurodegenerative conditions in rats.

Ischemia-reperfusion (I/R) damage is known for its hallmark of mitochondrial dysfunction [51,52]. Consequently, preserving mitochondrial activity is essential for the survival of neurons and neurological rehabilitation. The Nrf2/ARE signaling cascade is a regulator of mitochondrial biogenesis [53]. Nrf-2 interacts with mitochondrial transcription factor A (*TFAM*), which is a downstream target gene of peroxisome proliferator-activated receptor gamma coactivator 1-alpha (PGC-1α) and drives the transcription and replication of mitochondrial DNA [54]. According to Sandhu et al., the Nrf2/ARE and PGC-1α pathways interacted to promote mitochondrial biogenesis and neuroprotection [55]. During the dynamic mitochondrial process, fusion and fission constantly occur within mitochondria that continuously undergo morphological changes [56,57]. As one of the mitochondrial proteins, OPA1 regulates mitochondrial fusion at the inner mitochondrial membrane (IMM) [58]. A decrease in energy production and abnormal mitochondrial cristae remodeling are linked to OPA1 downregulation [59]. Upon I/R injury in rats, OPA1 expression was significantly downregulated in RGCs and ONs [29,60]. OPA1 appears to exert neuroprotective effects in the retina by modulating mitochondrial function [33]. Immunoblotting observations indicated that vitamin B3 promotes mitochondrial fusion after rAION treatment, as indicated by an increase in OPA1 expression (Figure 6). Based on the above results, vitamin B3 increases mitochondrial biogenesis via the upregulation of Nrf2 and promotes mitochondrial fusion via the upregulation of OPA1.

It has been established that the mitochondrial membrane is the location of the proapoptotic protein BAX, which is essential for mitochondria-mediated apoptosis [44]. The activation of BAX causes the permeabilization of mitochondrial membranes, which leads to the release of the proapoptotic effector cytochrome c and the development of apoptosis complexes as well as the induction of the caspase cascade [61]. The release of cytochrome c into the cytoplasm is one of many processes that lead to mitochondrial dysfunction [62]. Previous studies indicated that the activation of BAX-cytochrome c release induces apoptosis. As a result of the rAION induction, oxidative damage and apoptosis are observed. The mitochondrial-mediated regulation of cytochrome c release involves mediators such as BAX and cytochrome c in this apoptotic pathway [30]. It has been demonstrated that OPA1-mediated downregulation of BAX can alleviate the loss of RGCs caused by ischemia [63]. Furthermore, overexpression of OPA1 tightens and limits cytochrome c release, providing protection against the effects of ischemic brain injury after stroke [64]. A reduction in BAX and cytochrome c expression was observed in the immunoblot results after treatment with vitamin B3, effectively inhibiting apoptosis and reducing mitochondrial dysfunction. Consequently, vitamin B3 exerts antioxidative effects and reduces mitochondrial dysfunction.

**Figure 7:**
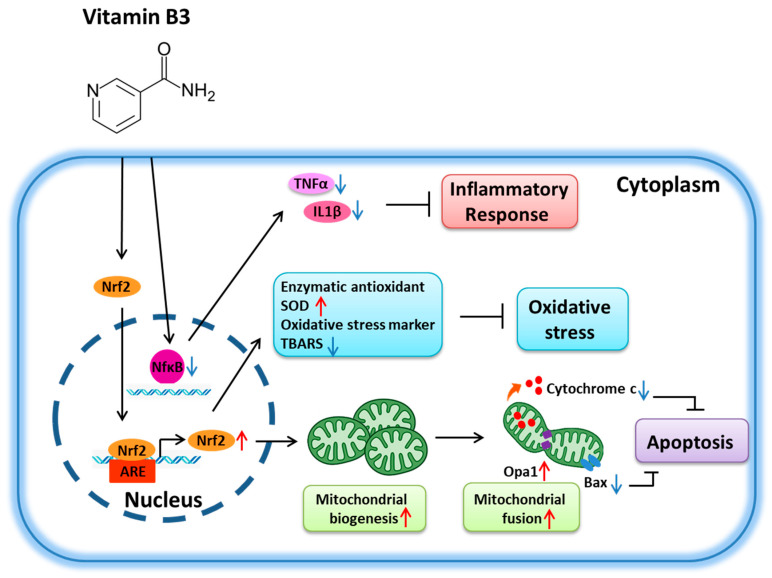
Summary of the neuroprotective effects of vitamin B3 in the rAION. Vitamin B3 treatment provided better anti-inflammatory, antioxidative, and antiapoptotic effects than PBS treatment.

Several studies have demonstrated that vitamin B3 has a favorable safety profile and high dose tolerability, and is affordable, making it easy to translate to small-scale clinical trials, and it also has an excellent history of long-term clinical use [65]. While lowering the expression of BAX and cytochrome c, vitamin B3 dramatically enhanced the expression of Nrf2 and OPA1 (Figure 7). Additionally, vitamin B3 dramatically reduces the activation of NF-κB caused by rAION, which in turn decreases the generation of inflammatory cytokines including IL-1β and TNF-α. There are many neuroprotective mechanisms implicated in response to rAION-induced retinal damage following pretreatment with vitamin B3, including Nrf2 for antioxidative activities and OPA1-BAX-cytochrome c for antimitochondrial apoptosis signaling pathways, as well as NF-κB for anti-inflammatory signaling.

## 5. Conclusions

In conclusion, vitamin B3 was found to act as a neuroprotective agent by promoting nerve regeneration and the functional recovery of RGCs. Besides activating Nrf2 pathways, inhibiting mitochondrial apoptosis, and reducing inflammation, it enhances the intrinsic regeneration capacity of RGCs after rAION. According to the available evidence, vitamin B3 may be helpful for treating AION in the clinical setting.

## Data Availability

The data presented in this study are available in the article.
